# P11 Improving the investigation and diagnosis of spontaneous bacterial peritonitis on the medical take

**DOI:** 10.1093/jacamr/dlac133.015

**Published:** 2023-01-19

**Authors:** Jack Stanley

**Affiliations:** University Hospitals Bristol and Weston, UK

## Abstract

**Objectives:**

To (i) improve the frequency of ascitic fluid sampling in decompensated cirrhotic patients; (ii) improve knowledge and confidence around ascitic fluid sample investigation and interpretation; and (iii) improve compliance with the decompensated cirrhosis bundle.

**Background:**

Cirrhotic patients are vulnerable to infection and can present with subtle clinical signs and laboratory results, making diagnosis of infection challenging. Spontaneous bacterial peritonitis is common in these patients and associated with a high mortality. BSG and BASL guidance suggests that all patients with ascites should have an ascitic tap performed on presentation to screen for infection, yet compliance with this directive is poor, leading to delays in diagnosis and poorly targeted antibiotic use. The reasons for this include lack of knowledge, lack of confidence in performing ascitic fluid sampling and lack of clarity on the samples required.

**Methods:**

We undertook a two cycle quality improvement project utilizing PDSA methodology. We focused both on practice and knowledge. Data collection – Identification of all decompensated cirrhotic on the medical take across a month via presenting complaint and discharge letter screening. Retrospective case note review was performed using a standardized proforma. Simultaneously junior doctor knowledge and confidence was assessed via three online questionnaires. Cycle 1 – Awareness building through posters and email. Dissemination of guidance around ascitic fluid sampling. Online education session provided for remote access (during COVID-19). Cycle 2 – Developed and implemented a new online order bundle for decompensated cirrhotic patients. Further awareness building in ED and medicine.

**Results:**

Three months of data collection were performed (included patients *n*=16, 15 and 14 respectively) and three rounds of knowledge surveys were undertaken (response *n*=40, 30 and 27 respectively). Ascitic tap attempts in appropriate patients rose from 50% to 83%. Requesting of (entirely) correct investigations for ascitic fluid increased minimally (67% to 75%). There was an increase in the percentage of those who classed themselves as somewhat or very confident (60 to 70% combined) regarding performance of ascitic tap . Despite this improvement in confidence, understanding of the correct testing and requesting procedure for ascitic fluid did not improve (96% incorrect). Compliance with the cirrhosis care bundle improved from 50% to 71%.

**Conclusions:**

Through our study we saw improved practice with regards to performing ascitic fluid sampling and utilizing the care bundle. Due to the design of our project we cannot say for certain that this was related to our intervention. Notwithstanding this we created digital resources in response to the COVID-19 restrictions and changed the order pathway for ascitic fluid sampling, making the process more intuitive and implementing sustainable, lasting change. We improved confidence without improving knowledge. This represents a further area for development as we take the project forwards. Our design does not allow us to comment upon patient outcome in response to our changes. Moving forwards our project shows the need to understand the barriers to junior doctors investigating patients with infections (such as lack of confidence and knowledge). The approaches used here could be utilized more widely to improve practice.

